# Advanced Procedure of Simultaneous Electrodeposition from a Natural Deep Eutectic Solvent of a Drug and a Polymer Used to Improve TiZr Alloy Behavior

**DOI:** 10.3390/ma16124387

**Published:** 2023-06-14

**Authors:** Manuela Elena Voicu, Florentina Golgovici, Mariana Prodana, Doina Draganescu, Ioana Demetrescu

**Affiliations:** 1 Department of General Chemistry, University Politechnica of Bucharest, Splaiul Independentei Street, No. 313, 060042 Bucharest, Romania; manuela_elena.voicu@upb.ro (M.E.V.); mariana.prodana@upb.ro (M.P.); ioana.demetrescu@upb.ro (I.D.); 2 Department of Pharmaceutical Physics and Informatics, “Carol Davila” University of Medicine and Pharmacy, 020956 Bucharest, Romania; doina.draganescu@umfcd.ro; 3 Academy of Romanian Scientists, 3 Ilfov, 050094 Bucharest, Romania

**Keywords:** TiZr alloy, gentamicin, NADES, electrochemical stability, drug release, antibacterial activity

## Abstract

This paper presents research about the embedding and release of gentamicin from an electrochemical deposition of polypyrrole from ionic liquids such as choline chloride on TiZr bioalloy. The electrodeposited films were morphologically investigated using scanning electron microscopy (SEM) with an EDX module, and polypyrrole and gentamicin were both identified using structural FT-IR analysis. The film’s characterization was completed with an evaluation of hydrophilic–hydrophobic balance, with electrochemical stability measurements in PBS and with antibacterial inhibition. A decrease in the value of the contact angle was observed from 47.06° in the case of the uncoated sample to 8.63° in the case of the sample covered with PPy and GS. Additionally, an improvement in the anticorrosive properties of the coating was observed by increasing the efficiency to 87.23% in the case of TiZr–PPy–GS. A kinetic study of drug release was performed as well. The drug molecule might be provided by the PPy–GS coatings for up to 144 h. The highest amount released was calculated to be 90% of the entire drug reservoir capacity, demonstrating the effectiveness of the coatings. A non-Fickian behavior was established as a mechanism for the release profiles of the gentamicin from the polymer layer.

## 1. Introduction

Since the last century, when Branemark discovered osseointegration [1,2], titanium’s importance was growing widely, being recognized soon as the golden standard for metallic implant biomaterials. Due to the need for enhanced mechanical properties, alloys with other metals such as Fe, Cu, Al, V, Nb, Cu, Ta, and Zr were elaborated, having different microstructures and leading to binary and tertiary alloys with improved properties, which were deeply investigated coated and uncoated [3,4,5,6]. When Zr alloys were more available in bioapplications [7], due to their decreased use in energy and electronics, the alloy TiZr which has remarkable mechanical properties, stability, and biocompatibility, was promoted as a Ti alternative [8]. Nowadays, in dentistry, this is used under the name Roxolid, containing 15–17% Zr, but more recent investigations propose an increased content in Zr implants, which is supposed to have better properties regarding mechanical and corrosion fields [9,10]. Nano level and nanotechnology, with their merits, introduced more performances as well [11,12] and permitted the embedding and release of various drugs such as ibuprofen, gentamicin, cysteine, and vancomycin [13,14,15]. Nowadays, when the number of surgical interventions is dramatically increasing due to the population aging with osteoporosis development, it is mandatory to find safe ways to reach surgery success, which is dependent on long-term implant stabilization and integration in the new bone-formed surrounding.

Frequently, post-operative infection occurs, requiring antibiotics to be administered to avoid the development of more aggressive health problems and long hospital time [16,17]. Impact evaluation of healthcare-associated infections on length of stay and costs of the treatments was an issue in the last years and was leading to more research and knowledge about the drugs able to solve the problem [15]. Moreover, considering that the strategy of infection prevention associated with dental and orthopedic implants require controlled drug release for a specific local therapeutic concentration, it is more important to know more about the bacteria inhibition and the host body response. In this context, metallic Ti drug-eluting implants such as TiZr with various drugs and agents able to deliver incorporated molecules were elaborated and studied. It is to mention conventional drug-eluting implants, anti-inflammatory and anti-resorptive drugs, antibiotics agents, and growth factors [18,19]. More recently, smart drug delivery systems able to respond to various stimuli such as heat, pH, light, and magnetic and electric fields [20,21] have been investigated as well. Special attention has been paid to gentamicin as probably the most used antibiotic on coated implants presented in the literature [22,23,24]. The extensive use of gentamicin (GS) is probably due to its effect in inducing mesangial cell contraction and filtration reduction. Cell biology tests with platelet-activating factor, calcium-sensing receptor (CaSR) stimulation, and reactive oxygen species (ROS) were able recently to put in evidence of such contractions [25]. Older investigations have demonstrated that calcium channel blockers in cell proliferation inhibition occur when a high level of free calcium is available to stimulate the phospholipases, proteases, and nucleases, which play a role in membrane disruption when more therapies are used in conjunction [22]. Based on such knowledge, gentamicin was nominated as the most used drug with an important antibacterial effect against ESKAPE pathogens [26].

This type of polymeric-gentamicin coating is very useful when applied in orthodontics to prevent periodontal problems in patients with improved difficulty in maintaining their oral hygiene [27] and can be very important in implantology to potentially diminish the rate of marginal bone loss around implants, in particular with different necks [28].

Drugs must be contained in a solvent during coating manufacture. Water is the most widely used and appropriate solvent for pharmaceutical substances. However, to improve the solubility of medications that are just slightly soluble in water, organic solvents such as ethanol, acetone, or ethers are sometimes required [29]. In light of this, achieving the ideal solvent selectivity while taking into account a number of variables, including the efficiency and productivity of a chemical process, is a tedious task. Due to this, a new class of natural deep eutectic solvents (NADES) has emerged as a viable and encouraging media substitute for an aqueous solution. NADES is environmentally friendly, economical, simple to prepare, and green substances [30].

With these facts in mind, the current study provides information on how NADES made using choline chloride as a hydrogen bond acceptor (HBA) and L-lactic acid (LA) as a hydrogen bond donor (HBD) in a 1:2 molar ratio can be used as an electrolyte for pyrrole polymerization. Gentamicin drug embedding and release is the subject of the present manuscript, but its deposition is an advanced new procedure simultaneously with a polymer from a natural deep eutectic solvent. The characterization of this co-deposition with polypyrrole, the electrochemical stability, and antibacterial effects complete the novelty of this manuscript and permit conclusions about electrodeposition performances.

## 2. Materials and Methods

### 2.1. Reagents

Ti50%Zr alloy, choline chloride (ChCl 99%—Sigma Aldrich, St. Louis, MI, USA), lactic acid, and gentamicin sulfate (GS) powder (Alfa Aesar, Thermo Fisher, Kandel, Germany) were used for natural deep eutectic solvent preparation. Pyrrole (Py) (98%—Sigma Aldrich), electropolymerized in ionic liquid.

A phosphate-buffered saline (PBS) of pH 7.4 with the following composition: 8 g/L NaCl, 0.2 g/L KCl, 1.42 g/L NaHPO_4_, 0.24 g/L KH_2_PO_4_ (Sigma-Aldrich), was used to electrochemical characterization of the coatings and drug release.

Luria–Bertani medium used for antibacterial effect: 10 g/L peptone, 5 g/L yeast extract, 5 g/L NaCl.

### 2.2. Substrate Coating Protocol

Prior to each experiment, the metallic substrate, TiZr alloy samples (20 mm × 20 mm × 2 mm), were ground mechanically using Buehler equipment with SiC grit paper up to #2500 size. After the polish, the samples were ultrasonically washed with water (10 min) and ethanol (10 min) to remove impurities from the surface.

A eutectic mixture of choline chloride–lactic acid in a molar ratio of 1:2 was heated to approximately 85 °C under continuous stirring so that a homogeneous solution was finally obtained. The liquid was cooled to room temperature, and 0.5 M Py was added. Additionally, 50 mM of GS was added with continuous stirring.

Electrochemical methods were used to collect data on the electropolymerization of PPy and PPy–GS coatings using NADES as electrolytes, including cyclic voltammetry and chronoamperometry. An AutoLab40 potentiostat/galvanostat (Radiometer Analytical SAS, Lyon, France) was used for all electrochemical polymerization. A single-compartment glass cell was utilized as the electrochemical cell, in which the working electrodes were TiZr alloy samples with a 1.8 cm^2^ surface area. The counter electrode was a high surface area platinum (Pt), while silver wires served as the quasi-reference electrode [31]. The samples were rinsed with ultrapure water and ethanol after the PPy or PPy–GS coatings had grown, and then they were allowed to air dry.

### 2.3. Surface Characterization

The morphology of the synthesized PPy and PPy–GS coatings surface was investigated using a scanning electron microscope (SEM) coupled with energy dispersive X-ray spectroscopy (EDX) (SU8230, HITACHI High-Technologies Corp., Tokyo, Japan) at 10 kV accelerating voltage. EDX is used to obtain the elemental composition of the deposition.

Their chemical structure was identified using Fourier-transform infrared spectroscopy (FTIR) from Perkin-Elmer, Shelton, WA, USA. Spectra were collected from 4000 to 600 cm^−1^ at a resolution of 4 cm^−1^ at 30 captures per sample.

By measuring the contact angle using PBS, the surface wettability characteristics were assessed. The average value of five measurements made on five distinct regions of the specimens using a CAM 100 compact contact angle meter from KSV Instruments (Espoo, Finland) was taken into account. A Hamilton syringe was used for the measurements, generating water droplets that were between 3 and 5 μL. For each sample, a minimum of three analyses were carried out, and the mean values are those that are shown. Microsoft Excel was used to compute the standard deviation during experiments that were conducted at room temperature.

### 2.4. Electrochemical Measurements

Using PBS as an electrolyte, the protective characteristics of the PPy and PPy–GS coatings electrosynthesized from NADES were also evaluated.

Open-circuit potential (OCP), as well as electrochemical impedance spectroscopy (EIS), and potentiodynamic polarization (Tafel plots), were carried out in an electrochemical cell with three electrodes, a 1.8 cm^2^ surface of the working electrode (TiZr uncoated and coated) was exposed in PBS electrolyte, a platinum (Pt) as a counter electrode, and a reference electrode was an Ag/AgCl. EIS measurements were performed at OCP with a 10 mV AC potential amplitude and a frequency range of 100 kHz to 50 mHz, and the data resulting from Nyquist and Bode plots were fitted using equivalent circuits. Potentiodynamic polarization curves were obtained at a sweeping rate of 2 mV s^−1^.

### 2.5. Release of Gentamicin

The TiZr–PPy–GS samples were immersed in 50 cm^3^ of PBS with a pH value of 7.4 medium release and investigated through a GS release was investigated using a UV–VIS spectrometer (model UV1720, UVISON Technologies Limited, London, UK) at different immersion periods. A volume of 3 mL was withdrawn from the medium release at different intervals of time and was replaced with the same volume of fresh PBS to keep the volume constant. The samples were analyzed at λ = 203 nm, and the cumulative drug release was calculated. The polymer coatings were rigorously cleaned with distilled water and dried before the drug release testing.

### 2.6. Antibacterial Effect

*Escherichia coli* (ATCC 25922), a Gram-negative coliform bacterium, and *Staphylococcus aureus* ATCC 6538, a frequently causing Gram-positive pathogen, were used to evaluate the antimicrobial activity of the samples. The turbidimetric method was used to take the measurements. Antibacterial tests were frequently performed by culture for 24 h at 37 °C in Columbia Agar with 5% sheep blood (Oxoid, Basingstoke, UK). After that, the sterile physiological solution was used to generate a bacterial suspension (inoculum), which was then adjusted using a densitometer to a MacFarland 0.5 up to a CFU count of 10^8^ CFU/mL.

In sterile polypropylene tubes, the three test samples—uncoated, with adsorbed material, and bonded—were immersed in bacterial suspensions. Sterile saline solution served as a negative control, and the inoculum was provided as a positive control. For 24 h, all recipients were left to incubate at 37 °C.

Equation (1) was used to determine the antibacterial activity after analyzing the absorbance of microbial cultures at 600 nm with an automated analyzer, the Chemwell 6010:

(1) $$I\left(\%\right)=\frac{\left(C_{24}-C_{0}\right)-\left(T_{24}-T_{0}\right)}{\left(C_{24}-C_{0}\right)}\times 100$$

where I is the growth inhibition index; C_0_ is the positive control’s optical density corrected for the blank at time 0; C_24_ is the blank-corrected optical density of the positive control after 24 h; T_0_ is the blank-corrected optical density of infected media in the presence of test samples; and T_24_ is the blank-corrected optical density of infected media in the presence of test samples at 24 h.

### 2.7. Statistical Analysis

To ensure their reproducibility and to statistically assess their importance, every measurement was examined at least in triplicate. All relevant information was presented with their standard errors. For each sample, roughly 50 unique particles were measured to extract the particle sizes from the high-magnification SEM pictures and run a statistical analysis to learn more about the morphological characteristics of the coatings. The average particle sizes were determined from the data plotted as histograms.

## 3. Results and Discussion

### 3.1. The Coating Preparation

**Figure 1:**
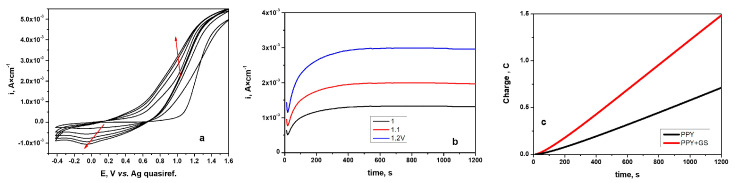
(**a**) Cyclic voltammograms on TiZr alloys recorded during PPy electrosynthesis from NADES electrolyte containing 0.5 M Py. The scan rate was 10 mV s^–1^; (**b**) chronoamperograms recorded at different applied potentials for electrosynthesis of PPy from NADES containing 0.5 M Py and 50 mM GS; (**c**) comparative charge–time plots obtained during electrosynthesis of PPy and PPy–GS at constant potentials of 1.2 V on TiZr alloy.

Investigating the growth of PPy layers on the TiZr alloys from NADES acting as electrolytes was the initial step of our study. The behavior of TiZr alloys was investigated using cyclic voltammetry in NADES made from choline chloride, lactic acid, and 0.5 M pyrrole monomer. Figure 1a illustrates voltammograms (CVs) obtained during PPy growth on biomaterial.

Figure 1a shows that we obtained the initial cycle in a different form from the subsequent recorded cycles. A shoulder anodic peak can be seen, which denotes the oxidation of the monomer or the beginning of polymer film generation.

It can be seen that the anodic peak current density associated with the electropolymerization of the pyrrole grows as the number of cycles increases, indicating a progressive increase in the electrical charge.

During the cathodic scan, the relevant reduction peaks can be observed. The current density also grows for these as the number of scan cycles rises. Because of its reduction from the oxidized to the neutral state, this can be correlated with the release of a small amount of chloride ion species from the polymer layer [32,33]. The two red arrows in Figure 1a show the increase in the value of the cathodic and anodic peak current density, respectively.

Following the establishment that NADES was able to be used as an electrolyte for the pyrrole electropolymerization on a TiZr substrate, the next step was to use chronoamperometry to synthesize films of either PPy or PPy with the drug incorporated. Figure 1b shows typical current-versus-time curves obtained for PPy–GS formation using NADES electrolyte at various applied potentials.

Figure 1c demonstrates that the polymerization charge rises when gentamicin is added to the electrolyte.

After electropolymerization, a dark, homogeneous, and adherent PPy or PPy–GS layer completely covered the TiZr alloys.

The chronoamperometry method was shown to yield the most reproducible values for PPy and PPy–GS growing on TiZr alloys at an applied potential of 1.2 V (vs. Ag quasi reference). These characteristics were developed after extensive research at various deposition times and applied potentials related to the prepared film quality, respectively.

For further investigations, the PPy and PPy–GS coatings on TiZr alloys were electrosynthesized using NADES electrolytes and achieved the same total charge value of 4 C.

### 3.2. Morphological and Structural Characterization of PPy and PPy–GS Coatings

#### 3.2.1. Scanning Electron Microscopy (SEM) Measurements

**Figure 2:**
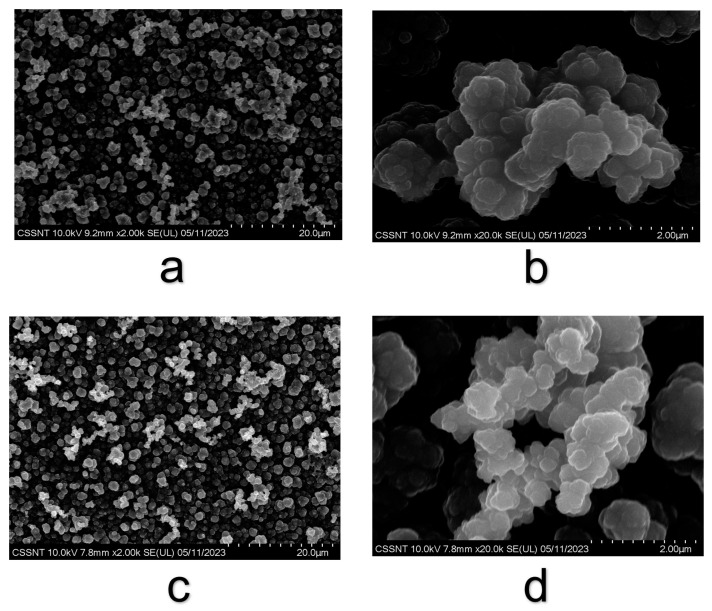
SEM micrographs at two magnifications (×2 k, ×20 k) for PPy (**a**,**b**) and PPy–GS (**c**,**d**) electrodeposited on TiZr alloy.

SEM-EDX analysis was used to evidence the influence of the electrodeposition parameters in composition, see Figure 2. The SEM images show that the supplied biomaterial developed a specific shape of PPy and PPy–GS coatings under potentiostatic control in a NADES-based electrolyte.

SEM micrographs of the electrochemically created polymeric layers on the TiZr alloy substrates are shown, which include cauliflowers structures typical for polypyrrole.

The granular surface morphology of PPy and PPy–GS was observable in the SEM micrographs. From the high-magnification SEM pictures, the average particle size was determined by counting about 50 particles per sample. When the drug is incorporated into a polymeric structure, and the PPy or PPy–GS films completely cover the alloy’s surface, the size of the clusters increases. The mean particle size was calculated as 0.2 ± 0.02 µm for PPy and 0.4 ± 0.03 µm for PPy–GS coatings.

Also, the SEM images suggest that no insoluble precipitates associated with drugs are visible on the surface of biomaterials.

This exemplifies how completely the drug was included in the PPy layer.

**Figure 3:**
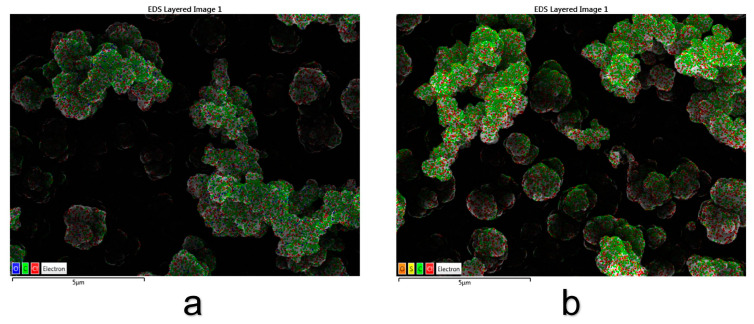
Mapping analysis for (**a**) PPy and (**b**) PPy–GS electrodeposited on TiZr alloy.

Figure 3 represents the elemental analysis-mapping of the PPy or PPy–GS films obtained by EDX analysis.

From the elemental analysis, for the PPy electrodeposited on TiZr alloy (Figure 3a), carbon and oxygen appear because of the polymeric deposition, and chlorine appears from the electrolyte solution (choline chloride). Titanium and zirconium do not appear in the elemental composition of the surface, which means that the film formed on the alloy surface is complete, homogenous, and without discontinuities.

In polymeric films containing gentamicin (Figure 3b), sulfur and oxygen appear because the drug contains sulfate ions in the chemical structure, a fact that proves the successful incorporation of the GS into the PPy film.

#### 3.2.2. FTIR Analysis

The qualitative analysis of the electrochemically produced PPy and PPy–GS films was performed using FTIR spectroscopy measurements.

**Figure 4:**
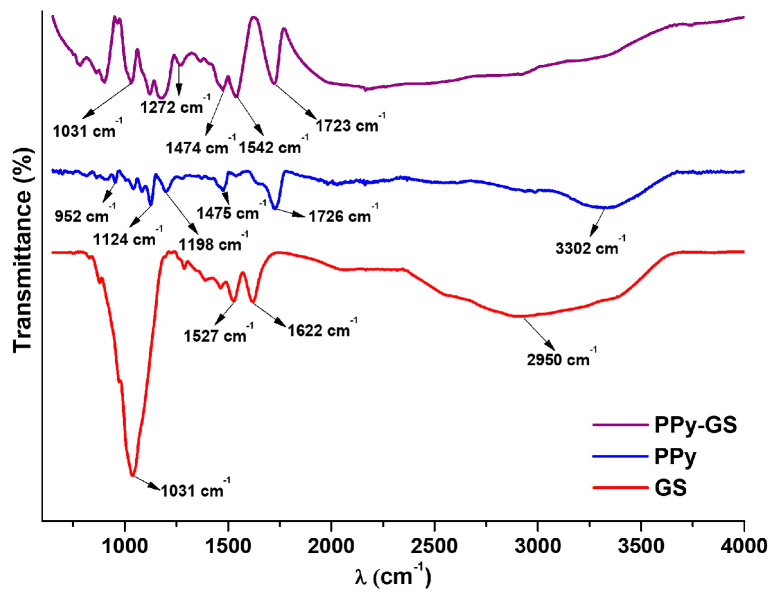
FTIR spectra of PPy and PPy–GS coatings electrochemically developed on TiZr alloy substrates using NADES electrolyte.

In the FTIR spectra presented in Figure 4, were found specific peaks of PPy and GS.

As could be seen, distinctive peaks may be found in the FTIR spectra of the alloys with polypyrrole coating. A wide shoulder being present in the 3400–3200 cm^−1^ area seen in Figure 4 serves as evidence for the N–H stretching vibrations of the pyrrole ring. While the band observed at 1475 cm^−1^ is attributed to the fundamental vibrations of the polypyrrole ring, those centered at 1198 cm^−1^ and the peak at 952 cm^−1^ can both be correlated with the C–N stretch [34,35,36,37].

For the GS spectrum, the peaks at 1622 cm^−1^ and 1527 cm^−1^ are assigned to the N–H vibrations of primary aromatic amines. The peak of 1031 cm^−1^ corresponds to the S–O group sulfur content [38].

In the PPy–GS spectrum, the presence of PPy is indicated by peaks at 1723 cm^−1^ and 1474 cm^−1^. Additionally, the shifted peaks or presence of peaks with lower intensity, characteristics of GS, due to the possible chemical interaction generated during the coated process suggests that the drug has been successfully incorporated into PPy film [39].

All these peaks indicate the presence of gentamicin in the polypyrrole structure.

#### 3.2.3. Contact Angle Measurements

The hydrophilic or hydrophobic properties of a biomaterial, which are normally determined by chemical composition and surface morphology, may be meaningfully highlighted by contact angle analysis.

Given that wetting phenomena affect cell adherence and proliferation on TiZr alloys coated with polypyrrole as well as those coated with polypyrrole and drug, contact angle values for uncoated and PPy- or PPy–GS-coated biomaterial were examined.

**Figure 5:**
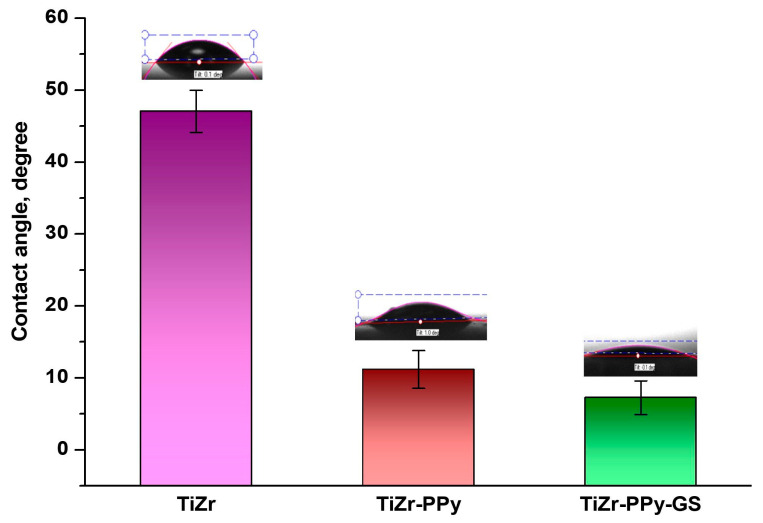
The contact angles of TiZr samples, PBS droplets.

The contact angle of the TiZr samples was measured by dropping PBS solution on different areas of the samples, and the average value was calculated. The average contact angle values were obtained from five measurements by dropping PBS solution on different areas of TiZr samples. These, with the corresponding standard error, are presented in Figure 5.

As we can see from Figure 5, the average value of the uncoated TiZr sample was 47.06° which is significant for a hydrophilic surface. Additionally, PPy film and PPy with GS, the contact angle decreased to 11.17°and 8.63°, respectively. Moreover, drug doping generated a small reduction in the contact angle value, indicating an improvement in hydrophilic characteristics. This fact is attributed to the relatively porous PPy–GS coating. Since a hydrophilic material allows the cells to attach, proliferate, and migrate appropriately, we can say that PPy or PPy–GS coatings are predicted to improve the biocompatibility of TiZr alloy [40].

### 3.3. Electrochemical Characterization

#### 3.3.1. Open-Circuit Potential Evaluation

**Figure 6:**
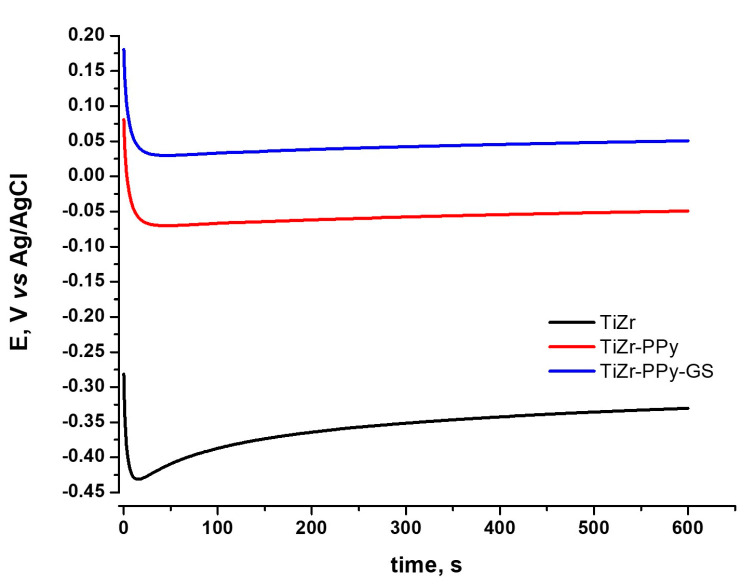
OCP behavior for coated and uncoated TiZr alloy in PBS, pH 7.4 at 37 °C.

Open-circuit potential (OCP) values were carried out for 600 s in PBS solution at pH 7.4 for uncoated and coated TiZr alloys with PPy and PPy–GS. From Figure 6, we can observe that, in the first seconds of immersion in the electrolyte, the potential values rapidly shift towards electronegative values for all the samples studied.

PPy- or PPy–GS-coated TiZr alloy recorded a significantly higher electropositive value of the stabilized potential than uncoated alloy, and the potential achieves a stable value quickly.

#### 3.3.2. Electrochemical Impedance Spectroscopy Tests

Electrochemical impedance spectroscopy is an efficient method for analyzing the kinetics of biomolecular interaction and a potent technique for examining the interfacial response mechanisms of modified electrodes [41]. Therefore, EIS is frequently used to examine chemical processes and changes in conductivity in an electrochemical circuit.

**Figure 7:**
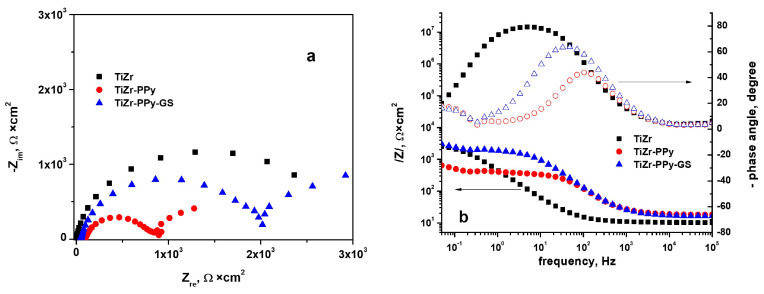
Nyquist (**a**) and Bode (**b**) diagrams for uncoated and Ppy- or Ppy–GS-coated TiZr alloy in PBS with pH 7.4 and 37 °C.

The spectra recorded at the open-circuit potential in PBS electrolyte, at 37 °C, for uncoated and PPy- or PPy–GS-coated TiZr alloy are presented as Nyquist and Bode diagrams in Figure 7.

As we can observe from Figure 7a, the Nyquist diagram recorded for uncoated biomaterials reveals one capacitive loop, while For the TiZr covered with polypyrrole containing or not embedded drug samples, two capacitive semicircles could be seen in the Nyquist plot. This fact could be explained by the development of two interfaces at the coating solution and alloy-coating, respectively. The capacitive semicircle diameters have a higher value in the case of PPy–GS coating.

From the Bode diagrams shown in Figure 7b, one can observe the appearance of a single-phase angle maximum for the uncoated TiZr alloy, while, as expected, two-phase angle maxima appear for the coated samples, one at high values and frequency averages, the other at low-frequency values.

The maximum phase angle value of 80 degrees for uncoated biomaterial indicates a capacitive behavior of the interface. It can also be observed that the inclusion of gentamicin in the polymer structure leads to an increase in the maximum value of the phase angle, indicating a shift towards a capacitive behavior of this interface.

**Figure 8:**
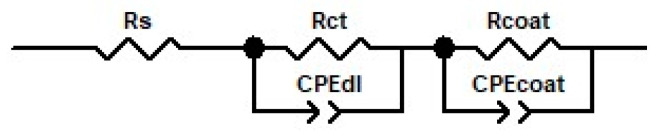
Electrical equivalent circuits proposed for fitting the experimental impedance spectra for uncoated and Ppy- or Ppy–GS-coated TiZr alloy.

As illustrated in Figure 8, the electrical equivalent circuit (EEC) and ZView 2.9 software (Scribner Associates Inc., Southern Pines, NC, USA) were used to examine the experimental impedance data. The values computed for the suitable electrical circuit elements are shown in Table 1. A value of chi-squared (2) of approximately 10^−4^ was identified when the experimental data were fitted, indicating that the fitting errors were minimal.

For uncoated TiZr alloy, a basic Randles circuit was formed of the electrolyte resistance (Rs), and to explain the electrolyte–substrate contact, a parallel combination of double-layer capacitance (CPE_dl_) and charge transfer resistance (R_ct_), was used.

Because it was assumed that a second interface would occur in the case of coated samples based on the design of the Nyquist and Bode diagrams, in addition to the Randles circuit, a second parallel combination was added. This was related to the coating capacitance (CPE_coat_) and coating resistance (R_coat_) at the polymeric coating–solution interface.

The acquired fitting parameters reveal high values of the coating resistance R_coat_ and low values of the capacitance component of CPE_coat_, values that illustrate the strong capacitive behavior of the coatings, and these values are shown in Table 1. The CPE–P independent parameter displays values near one, indicating that the studied samples’ have near-ideal capacitors behavior. The higher values for coating resistance are obtained for the polymeric coating with embedded gentamicin, indicating that it offers the best anti-corrosion protection in PBS electrolyte.

#### 3.3.3. Biocorrosion Tests

A third method used for the electrochemical characterization of the studied samples was the potentiodynamic polarization at a low rate of the potential scan.

**Figure 9:**
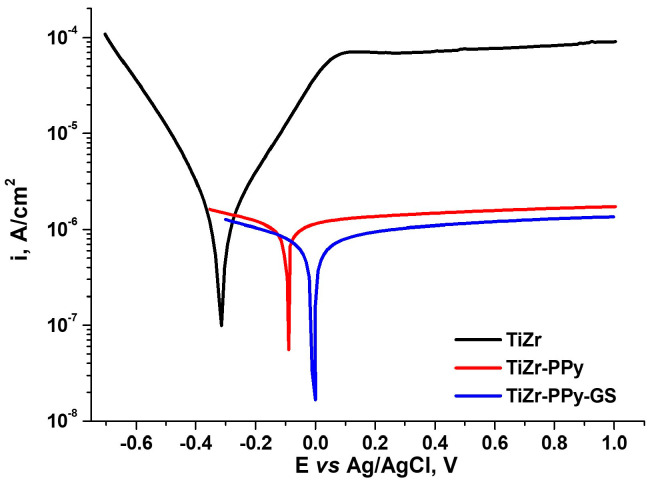
Polarization curves recorded for uncoated and PPy- or PPy–GS-coated TiZr alloys in PBS.

From Figure 9, we can observe a shift towards more electropositive values of the corrosion potential of the coated biomaterial compared to the uncoated one, as well as a decrease in the corrosion current density, indicating that the polymer coating has an anticorrosive protective effect.

The Tafel slope extrapolation method and the polarization resistance method were used to obtain the corrosion kinetic parameters from the polarization curves shown in Figure 9. Several kinetic corrosion characteristics, including corrosion potential (E_corr_), corrosion current density (i_corr_), gravimetric index (k_g_), corrosion rate (CR), and polarization resistance (R_P_), are expressed by the values in Table 2. The values of the corrosion current density obtained using the two methods were equivalent. The Stern–Geary equation was used to calculate the polarization resistance, denoted as Rp [42], which can indicate the level of corrosion protection. The higher the polarization resistance value is obtained, the lower the value of the corrosion rate will be.

According to the ASTM-G59 standard, corrosion current density (i_corr_) and corrosion rate (CR) were calculated [43]. The analyzed PPy- or PPy–GS-coated TiZr samples exhibit higher polarization resistance compared to uncoated alloys, demonstrating a decrease in the corrosion rate of each of the investigated biomaterials in PBS. The findings of the statistical analysis of all parameters are presented as mean ±1 standard deviation.

Based on the electrochemical parameters obtained, the protective efficiency, PE (%), has been evaluated quantitatively using Equation (2).

(2) $$\mathrm{PE}\left(\%\right)=\left[1-\left(\frac{i_{\mathrm{corr}}}{i_{\mathrm{corr}}^{0}}\right)\right]\times 100$$

where i_corr_ and $i_{\mathrm{corr}}^{0}$ are the corrosion current density of the PPy- or PPy–GS-coated TiZr and uncoated TiZr alloy, respectively.

In conclusion, it can be stated that covering the TiZr alloy with PPy–GS layers demonstrates higher corrosion protection than coating it with PPy layers. This agrees with the electrochemical impedance spectroscopy’s findings. Both coating types provide corrosion protection for studied biomaterial, although anodic reaction prevention is more important. The best coating protective efficiencies, or capacity to prevent corrosion, were demonstrated by lower corrosion current densities and lower corrosion rates, as well as higher polarization potentials and higher polarization resistances.

### 3.4. Drug Release from the Polypyrrole Matrix

**Figure 10:**
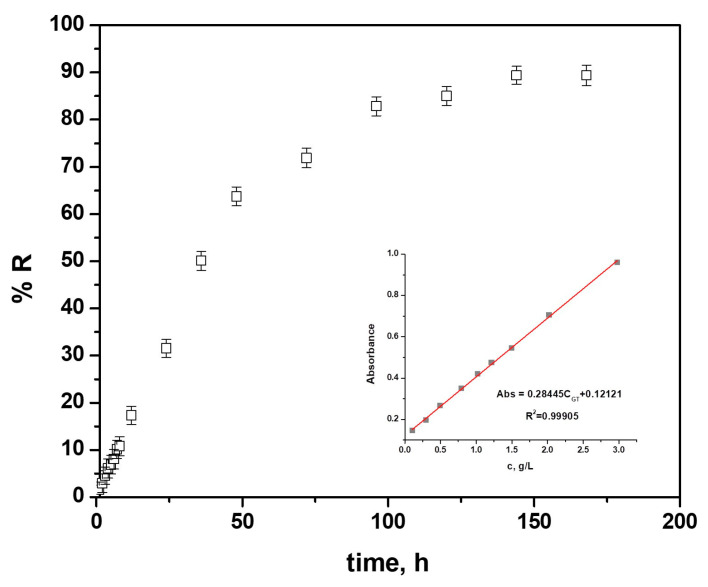
Gentamicin release profiles from the PPy–GS coatings electrosynthesized on TiZr alloy from NADES electrolyte containing 0.5 M Py and 0.05 M GS; inset: calibration curve recorded in PBS for GS (*n* = 3).

The samples of TiZr covered with polypyrrole and gentamicin were immersed in PBS solution to determine the release of the drug from the polymeric films. The calibration curve presented in Figure 10 was performed at 203 nm to determine the linear regression. As we can see, the obtained line equation was y = 0.28445x + 0.12121, and the correlation coefficient was r^2^ = 0.9991, indicating a significant linear regression (*p* < 0.0001), thus that the drug release from the PPy matrix might be regarded as suitable for this medium.

The initial concentration of gentamicin in the polymer coating was obtained using the same analytical method, and data analysis was performed using an average of three observations.

The drug release profile from polymer coatings electrosynthesized on TiZr alloys using a NADES based on choline chloride–lactic acid containing 0.5 M Py and 0.05 M GS is shown in Figure 10. 

The cumulative drug release, designated RE, is the fraction (percentage) of the active species released at time t in PBS, determined with Equation (3), as

(3) $$\mathrm{RE}=\frac{q}{q_{0}}\times 100$$

where q_0_ is the initial drug content of the coating, and q is the amount of drug released at time t.

As illustrated in Figure 10, an initial burst release effect up to about 18% during the first 12 h may be noticed for the PPy–GS coatings, with half the amount of drug incorporated in the polymer coating being released in about 48 h, followed by a prolonged release phase for the next 100 h and then a relatively constant release profile up to 144 h.

In only 2 h after immersion, the lowest effective gentamicin sulfate concentration against many bacteria, which is around 4 mg/L [24], was achieved.

As previously said, PPy–GS electrochemically generated coatings may be able to deliver drugs for longer periods. The cumulative drug released fraction after 144 h was in the region of 90%. The result obtained is in agreement with those obtained for other nanostructures formed on the surface of the TiZr alloy, having drug embedded [23,24]. Generally speaking, the diffusion of active chemicals toward the solution may be impacted by the morphological characteristics of the films [44]. Last but not least, various mathematical models (Korsmeyer–Peppas, Higuchi, Hixson–Crowell, Baker–Lonsdale, zero order, and first order) were used to analyze the gentamicin release from the PPy coatings data. The correlation coefficients used to fit the Korsmeyer–Peppas model (Equation (4)) to the experimental release data for all PPy–GS coatings were very close to 1 (r^2^ = 0.9988), indicating that the model can adequately characterize drug release.

A straightforward semi-empirical Equation (4), which is extensively employed in pharmaceutical research to quantify release kinetics [45,46,47], serves as the foundation for the Korsmeyer–Peppas model, also known as the power law model.

This equation is expressed as

(4) $$\mathrm{RE}={\mathrm{kt}}^{n}$$

where RE is the cumulative drug release percentage at time t, n denotes the diffusional exponent describing the drug release process, and k is a parameter that includes the structural and geometric properties of the drug/polymer system. When n is utilized to distinguish between various drug release mechanisms, the Korsmeyer–Peppas model may be viewed as a decision parameter to identify drug transport pathways.

The drug release from PPy coatings electrodeposited on CoCr alloys had a release coefficient of n > 0.5, which points to a non-Fickian behavior that also takes into account the polymer chain’s erosion.

The obtained value for diffusional exponent (n) for the PPy–GS coatings electrosynthesized on the TiZr alloy substrate was 0.516, indicating a non-Fickian behavior which also takes into consideration the erosion of the polymer chain [44,45].

The kinetic constant (k) value of 1.347 h^−1^ suggests a relatively fast release of the drug from the polymeric coatings.

### 3.5. Coating’s Antibacterial Characteristics

**Figure 11:**
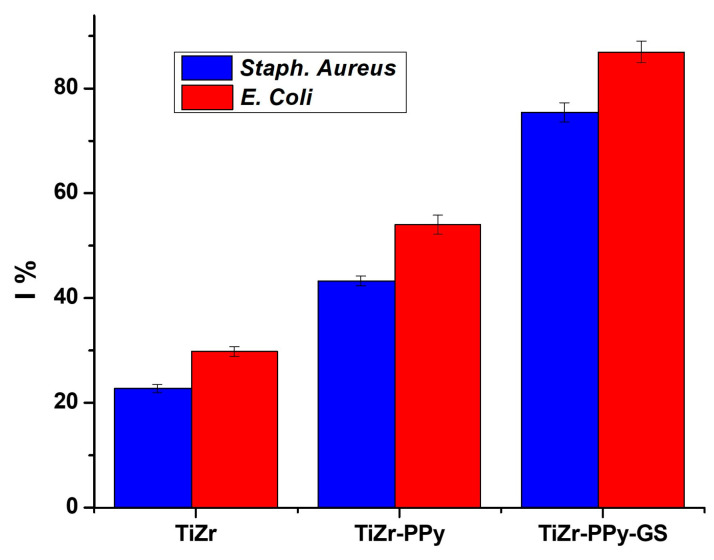
The bacterial inhibition levels.

For the two investigated bacteria, *Escherichia coli* and *Staphylococcus aureus*, the bacterial growth inhibition ratio (I%) are shown in Figure 11 for all three cases examined: uncoated samples or coated with PPy or PPy–GS. As you can see, the coating significantly boosts the samples’ antibacterial activity.

Because Gram-positive bacteria are more drug-responsive than Gram-negative bacteria due to the absence of the outer membrane, the bacterial inhibition level for *Staph. aureus* is, as can be seen, slightly lower than the value for *E. coli*.

For the PPy–GS-coated TiZr alloy, the obtained values for the inhibition index are 75.4 for *Staphylococcus aureus* and 86.9 for *Escherichia coli*, being higher values than those obtained for other nanostructures with embedded drugs [14,23]. Frequently better results are obtained for *Staphylococcus aureus* vs. *E. coli*, and this fact is attributed to differences in the membranes of the two organisms. The *Staph. aureus* has a multilayered outer peptidoglycan membrane, whereas the Gram-negative bacteria *E. coli* has two outer-layer membranes with only one layer of peptidoglycan. We conclude that GS embedding associated with a hydrophilic contact angle value supports an antibacterial effect due to the fact that it simultaneously inhibits bacterial attachment and biofilm development.

## 4. Conclusions

For the first time, on TiZr alloys, simultaneous gentamicin embedding during polypyrrole electrosynthesis from NADES electrolyte has been achieved.

The SEM micrographs for PPy and PPy–GS showed granular surface morphology and no signs of drug-molecule insoluble precipitates. The size of clusters grows when the drug is embedded into a polymeric structure, and the PPy or PPy–GS films completely cover the alloy’s surface. Recorded FTIR spectra have proven that gentamicin is present in the polypyrrole structure.

By monitoring the contact angle values, it was possible to establish that studied TiZr alloys had improved hydrophilic properties after being coated with PPy or PPy–GS.

Additionally, by covering the TiZr alloys with polymeric films or polymeric films containing gentamicin, the anticorrosive characteristics in PBS have been improved.

The PPy–GS coatings could provide the drug molecule for a prolonged period of time, up to 144 h, according to gentamicin release experiments. Half of the drug quantity embedded into the polymeric coating during polymerization is released in about 48 h. The polypyrrole layers served as an efficient drug reservoir, as seen by the greatest amount released, which was estimated to be about 90% of its total capacity. As a mechanism for the release profiles of the gentamicin from the polymer layer, a non-Fickian behavior was established.

Surface characteristics of the coating, as shown by SEM and wettability studies, are related to its antibacterial effects. Simultaneously embedded gentamicin sulfate during pyrrole electropolymerization using NADES electrolyte on TiZr alloy leads to more hydrophilic PPy–GS coating than uncoated TiZr, making it a potential material for biological applications as well as a promising candidate for reducing the post-surgical time of implantable TiZr alloy.

To better sustain PPy–GS coating as a candidate in reducing the post-surgical time of implantable TiZr alloys, in a future paper, our intention is to test the effect of other aggressive bacteria.

## Figures and Tables

**Table 1:** The values of EEC elements for coated and uncoated TiZr in PBS.

Sample	R_s_, Ω × cm^2^	R_coat_, Ω × cm^2^	CPE_coat_–T, µF × cm^−2^	CPE_coat_–P	R_ct_, Ω × cm^2^	CPE_dl_–T, mF × cm^−2^	CPE_dl_–P	Chi-Squared (χ^2^)
TiZr	10.6	-	-	-	2770	0.4	0.911	5.4 × 10^−4^
TiZr–PPy	15.4	781.6	14.7	0.858	713.6	4.07	0.98	3.4 × 10^−4^
TiZr–PPy–GS	18.3	1924	21.8	0.891	1390	2.09	0.97	1.7 × 10^−4^

**Table 2:** Kinetic corrosion parameters for uncoated and PPy- and PPy–GS-coated TiZr alloys in PBS.

Sample	*Tafel Method*				*Polarization Resistance Method*		
	E_corr_, mV	i_corr_, µA × cm^−2^	Kg, g × m^−2^h^−1^	CR, µm × Year^−1^	R_P_, KΩ	i_corr_, µA × cm^−2^	PE, %
TiZr	−0.295 ± 0.03	2.75 ± 0.02	0.0261 ± 0.001	8.44 ± 0.07	17.8 ± 0.07	2.62 ± 0.04	-
TiZr–PPy	−0.097 ± 0.01	0.603 ± 0.01	0.0057 ± 0.0003	1.85 ± 0.03	71.5 ± 0.1	0.608 ± 0.01	78.07 ± 0.02
TiZr–PPy–GS	−0.006 ± 0.001	0.351 ± 0.01	0.0033 ± 0.0001	1.08 ± 0.01	108.75 ± 0.3	0.357 ± 0.005	87.23 ± 0.01

## Data Availability

The data presented in this study are available upon request.
